# Phosphoinositide-3-Kinase Signaling in Human Natural Killer Cells: New Insights from Primary Immunodeficiency

**DOI:** 10.3389/fimmu.2018.00445

**Published:** 2018-03-07

**Authors:** Emily M. Mace

**Affiliations:** ^1^Department of Pediatrics, Baylor College of Medicine, Center for Human Immunobiology, Texas Children’s Hospital, Houston, TX, United States

**Keywords:** phosphoinositide-3-kinase signaling, primary immunodeficiency, natural killer cell biology, human natural killer cells, natural killer cell development, natural killer cell cytotoxicity

## Abstract

Human natural killer (NK) cells play a critical role in the control of viral infections and malignancy. Their importance in human health and disease is illustrated by severe viral infections in patients with primary immunodeficiencies that affect NK cell function and/or development. The recent identification of patients with phosphoinositide-3-kinase (PI3K)-signaling pathway mutations that can cause primary immunodeficiency provides valuable insight into the role that PI3K signaling plays in human NK cell maturation and lytic function. There is a rich literature that demonstrates a requirement for PI3K in multiple key aspects of NK cell biology, including development/maturation, homing, priming, and function. Here, I briefly review these previous studies and place them in context with recent findings from the study of primary immunodeficiency patients, particularly those with hyperactivating mutations in PI3Kδ signaling.

## Introduction

### Human Natural killer (NK) Cell Development, Lytic Function, and Migration

Human NK cells are derived from bone marrow precursors and mature in the peripheral tissues, particularly the secondary lymphoid tissue (1–3). Their development can be defined by select cell surface receptor and transcription factor expression in combination with increasingly restricted lineage potential of developmental intermediates (4). Despite an increasing understanding of the relationship between NK cells and other innate lymphoid cell subsets, however, the exact nature of the steps of NK cell development is incompletely understood. This likely reflects the importance of the local microenvironment in tuning NK cell development and plasticity of NK cell developmental intermediates, as discrete tissue sites have unique resident NK cell populations (1).

Within peripheral blood, human NK cells comprise approximately 10% of lymphocytes and are broadly classified as CD56^bright^ or CD56^dim^, two subsets with distinct phenotypic and functional properties (5). CD56^bright^ are considered less mature than the CD56^dim^ subset, and their lesser frequency within peripheral blood is converse to their predominance in the secondary lymphoid tissue, where they are thought to develop (6). CD56^dim^ NK cells are cited as having the greatest capa-city for lytic function; however, similar capacity for lytic function can be elicited from CD56^bright^ NK cells with cytokine priming or activation (5, 7–10). CD56^bright^ cells are strong producers of cytokines, particularly IFNγ and TNFα and are frequently considered more immunoregulatory than CD56^dim^ NK cells. In addition, CD56^dim^ NK cell subsets can be further dissected to terminally mature subsets that can include adaptive NK cells with memory-like function for rapid response to previously encountered antigen (11–17).

Natural killer cell lytic function is mediated through the formation of an immunological synapse, a specialized signaling platform that directs the secretion of specialized secretory lysosomes containing perforin and granzymes (18, 19). Many of the steps leading to the formation of an NK cell lytic immunological synapse are common to other immunological synapses, including T cell synapses (20). There are also distinct features of the NK cell immunological synapse that are likely a function of unique mechanisms of NK cell activation and sensing, as NK cells use germline-encoded activating and inhibitory receptors to integrate signals that can lead to lysis of non-self, stressed, transformed, or virally infected cells (21). While an in-depth description of this process is beyond the scope of this review, key events in this process include firm adhesion to a target cell, actin polymerization and reorganization at the immunological synapse, lytic granule convergence, microtubule-organizing center (MTOC) (and lytic granule), polarization toward the synapse, granule exocytosis, and termination of the immunological synapse following target cell death (22). Relative to lytic synapses formed by cytolytic effector CD8^+^ T cells, NK cell lytic synapses are less rapidly formed and seemingly have a greater number of regulated steps to cytotoxicity, likely due to their non-antigen-restricted mechanism of sensing target cells and “missing-self” recognition (21). Their capacity for autologous killing is restrained by a process termed licensing, in which NK cells are licensed for lytic potential through the engagement of inhibitory receptors with self-MHC class I (23–25). While the molecular mechanisms of licensing are not fully understood, this process is reversible, and even unlicensed cells ultimately have the potential to be fully lytic (26–29). Through an understanding of licensing, priming, and cytokine-induced memory, it seems that NK cell function can be tuned for responsiveness through multiple mechanisms.

While less understood at a molecular level than immunological synapse formation, NK cell migration and homing are intrinsic components of both development and function. The accepted paradigm of NK cell development suggests that NK cells enter circulation to traffic to tissue at both an early develop-mental stage and ultimately as terminally mature cells. Intravital imaging of mouse lymph nodes demonstrates that NK cells patrol the T cell zone and make transient, yet direct, contacts with dendritic cells and T cells (30–32). This significant migratory behavior has obviously not been directly visualized in human tissue; however, human NK cells undergo spontaneous migration on developmentally supportive stromal cells (33). This intrinsic migratory capacity can be recapitulated by the differentiation of NK cells *in vitro*, and developmental intermediates have intermediate migratory phenotypes (33, 34). In the context of lytic function, NK cell migration plays a key role in the serial killing capacity of activated cells, which often kill up to 10 sequential targets (35–40). Activated NK cells have a more dynamic migration, with modes of migration that are distinct from resting cells (40). This includes a more motile scanning of targets, a specifically greater directional persistence, less time spent in arrest, and a greater migration speed. These changes in migration mode have been described between resting and cytokine-activated (40), or unlicensed and licensed human NK cells (39), suggesting a link between multiple forms of activation and changes in migratory phenotype; however, the mechanism by which these changes are induced is unknown.

Human primary immunodeficiency is a powerful model to determine the requirements for human NK cell function and development. In particular, studies of patients with Wiskott–Aldrich syndrome (41, 42), MyH9-related disorders (43, 44), DOCK8 deficiency (45), and Coronin 1A deficiency (46) have led to the definition of these as critical mediators of NK cell immune synapse formation and function and defined the molecular basis of their function in a uniquely human setting. A similar approach can be taken to determine the requirements for human NK cell development through the study of patients with NK cell deficiency as a result of loss of NK cells or NK cell subsets in peripheral blood [reviewed in Ref. (47, 48)]. In particular, a decreased frequency of the CD56^dim^ subset has been used as a readout for the impaired terminal maturation of NK cells. Using this approach, unexpected requirements for the eukaryotic DNA helicase complex components MCM4 and GINS1 specifically in NK cell development have been identified (49–51). Similarly, biallelic mutations in *IRF8* lead to specific loss of the CD56^dim^ subset, with accompanying severe viral susceptibility in affected patients (52), and *RTEL1* mutations can lead to the absence of NK cells in peripheral blood (53, 54). More puzzling are mutations in *GATA2*, which lead to specific loss of the CD56^bright^ subset with variable effects on absolute NK cell frequencies, although this effect may be due to depletion of all but adaptive NK cells in affected patients (55–58).

Cases of isolated NK cell deficiency, in which NK cells are the primary or only affected immunological subset, are relatively rare. However, the extreme susceptibility of these patients to viral infections, particularly of the herpesvirus family, underscores the importance of NK cells in human health and disease (47, 48). Much more common are primary immmunodeficiencies that may include deregulated NK cell function or phenotype as partof their spectrum of disease. These can be illustrative of requirements for NK cell development or function, despite involvement of other immune compartments. These primary immunodeficiencies that affect NK cells range from severe combined immune deficiency as a result of *IL2RG* (59) or *JAK3* (60) mutation, which defines the requirement for common gamma chain cytokine signaling in human NK cell development, to diseases including STAT1 gain-of-function (GOF) mutations (61) and STAT3 deficiency (62). In each of these cases, it is important to consider that other affected immune compartments can also impact NK cell phenotype and function. It can also be difficult to delineate between primary immmunodeficiencies that seemingly lead to a “hard stop” in NK cell maturation, such as MCM4 and GINS1 deficiencies and those that deregulate specific receptor expression or aspects of homeostasis, such as STAT1 GOF mutations. Regardless, in each case, the phenotype of deregulated NK cell development is accompanied by an effect on NK cell function that translates to susceptibility to infection and, in some cases, malignancy. Moving forward, however, it will be important to recognize these distinctions through the careful definition of what truly phenotypically and functionally defines NK cells and their subsets. In addition, determining the NK cell-intrinsic component to these mutations, such as by cell line modeling, is important for the proof of concept to define a particular gene as being required for human NK cell function.

The phosphoinositide-3-kinase (PI3K)-signaling axis plays a key role in a multitude of cellular functions. It is increasingly being recognized for its importance in the control of inflammation and cancer and is a particularly exciting target for new small molecule inhibitors designed to modulate its key players. Given its ubiquitous expression, perturbations in this pathway are predicted to impact a number of cellular functions. However, there are specific requirements for PI3K signaling in NK cell function, the importance of which are underscored by model organisms and recently described human mutations in *PIK3CD* that lead to significant defects in NK cell maturation and function (Table 1).

**Table 1:** Effect of phosphoinositide-3-kinase (PI3K) mutations relevant to activated PI3K delta syndrome on natural killer (NK) cell development and function.

Gene (protein)	Mutation type	NK function	NK number/phenotype	Reference
**Human**				
*PIK3CD* (p110δ)	GOF	Impaired	Decreased/affected	(63–67)
*PIK3CD* (p110δ)	LOF	ND	ND	(68)
*PIK3R1* (p85α)	GOF	ND	Decreased/ND	(69)
*PIK3R1* (p85α)	LOF	ND	Decreased	(70)
*PTEN* (PTEN)	LOF	ND	Decreased/ND	(71–73)
*PTEN* (PTEN)	OE	Decreased	ND	(74)

**Mouse**				
*Pik3cd* (p110δ)	Deletion	Impaired	Decreased/affected	(75, 76)
*Pik3cd* (p110δ)	Inactive	Impaired	Decreased/affected	(77)
*Pik3r1* (p85α)	Deletion	Impaired	Decreased/affected	(78)
*PTEN* (PTEN)	Deletion	Impaired	Increased/affected	(79)
*Inpp5d* (SHIP-1)	Deletion	Decreased (cytokine)	Decreased/affected	(80)
*PTEN* (PTEN)	OE	Decreased	Unaffected	(74)

*ND, not determined; GOF, gain-of-function; LOF, loss of function; OE, overexpression*.

### PI3K Signaling in Human NK Cells

Class IA PI3K are heterodimeric enzymes that consist of a regulatory p85 subunit and a catalytic p110α, -β, or -δ subunit; class 1B PI3K is composed of the p110γ subunit and p101 or p84 regula-tory subunit. While all PI3K isoenzymes play a key role in catalyzing the production of PtdIns(3,4,5)P3 from PtdIns(4,5)P2, evidence suggests that they have unique functions (81). In addition, while p110α and p110β are widely expressed, p110γ and p110δ expression is primarily restricted to lymphocytes. Activating signaling leads to PI3K-mediated generation of PtdIns(3,4,5)P3, the accumulation of which in the cell membrane provides a platform for pleckstrin homology (PH)-domain-containing proteins, including AKT, phosphoinositide-dependent kinase-1 (PDK1), and Tec family kinases such as BTK. This pathway is additionally regulated by phosphatases including phosphatase and tensin homolog (PTEN) and SH2-containing inositol phosphatase 1 (SHIP-1).

In human NK cells, the PI3K-signaling pathway plays a direct role in signaling downstream from activating receptors, including 2B4 and KIR receptors (82–84). The recruitment of p85, in combination with Grb2, is also necessary and sufficient for the propagation of signaling, leading to cytotoxicity downstream of NKG2D ligation association with the non-ITAM containing DAP10 adaptor (85–88). PI3K activity following recruitment to membrane proximal receptors leads to the production of PtdIns(3,4,5)P3 and the subsequent recruitment of PH domain-containing proteins such as PLCγ1, PLCγ2, Vav1, and Tec kinases. Antibody-dependent cellular cytotoxicity (ADCC) is mediated by PI3K- and ITAM-dependent signaling by CD16 through FcγR and/or TCRζ (89); PI3K signaling plays an additional role following FcγR ligation by activating ADP-ribosylation factor, which leads to PtdIns(4,5)P2 production by PI5K and phospholipase D activation (90).

Phosphoinositide-3-kinase activation and subsequent recruitment of PLCγ1 and PLCγ2 leads to mobilization of intracellular Ca^++^ stores. In addition, PI3K activates a Rac1–MEK–ERK pathway that is a key signaling pathway for actin reorganization and cellular polarization (91). The central role of PI3K in mediating cell polarization can be defined by its control of Cdc42 activation at the NK cell immune synapse; in particular, p85α acts as a scaffold to target and position PI3K and subsequently recruit guanine nucleotide exchange factors to the membrane (92). As such, the role of PI3K signaling in cytotoxicity and NK cell migration can be through the control of actin remodeling, polarization, and even granule exocytosis, which requires intracellular calcium store mobilization.

In addition to activating for cytotoxicity, PI3K plays a pivotal role in both priming and signaling downstream of cytokine activation. It is particularly important for the attenuation of signaling through IL-15, the critical NK cell development and survival cytokine (93). The activation of PI3K following IL-15 receptor ligation leads to the production of PtdIns(3,4,5)P3 and the recruitment of AKT to the cell membrane. AKT activation leads to survival and proliferation through the inhibition of pro-apoptotic Bcl2- and PDK1-dependent activation of mTOR, which promotes translation directly through the phosphory-lation of S6 kinase and the initiation factor eIF4E-binding protein (94). In mice, PI3K-dependent PDK1 activation *via* IL-15 signaling may also directly help to direct NK cell lineage commitment through the induction of E4BP4 and Eomes, and PDK1-deficient mice have loss of NK cell cellularity and function (95).

The critical role of PI3K in JAK–STAT signaling makes it key in potentiating the effects of cytokine priming, in which the threshold for NK cell activation is lowered by stimulation with common gamma chain cytokines (IL-2, -15, -21) or IL-12 and IL-18 (96–98). The therapeutic potential of cytokine priming is highlighted by recent studies of human memory-like NK cells with enhanced lytic function that can be generated by cytokine priming and can be reactivated after even extended periods of rest (99–101). These cells are of extreme interest for immune therapy and also highlight the importance of cytokine priming in generating NK cells that can rise to further challenge (102). Physiologically, priming leads to an increased antitumor effect of NK cells, including an increased production of cytotoxic effec-tor molecules, an increased conjugate formation with target cells, and an increased baseline activation of integrins (10). Interestingly, this effect in humans is primarily mediated by the CD56^bright^ NK cell subset, as opposed to CD56^dim^ NK cells, which are traditionally considered the more cytolytic subset. Small molecule inhibition of the PI3K-signaling pathway blocks this priming effect and attenuates the antitumor response, underscoring its importance in modulating NK cell function (10, 103). The importance of the PI3K-signaling pathway in NK cell priming and its implication in NK cell licensing (104) underscore its importance as a master regulator of NK cell-functional capacity.

Finally, PI3K signaling is required for NK cell chemotaxis to chemokines including CC chemokine ligand (CC)L2, CCL5, CXCL10, and SDF1α (105). While lymphocyte migration is thought to be mainly controlled by p110γ, p110δ is required specifically for chemotaxis mediated by the G-protein-coupled receptor sphingosine 1-phosphate receptor 5, which plays a key role in NK cell tissue localization (106, 107). Both p110δ and p110γ are activated for chemotaxis to CXCL12 and CCL3 and mediate NK cell migration to tissue and to the uterus during pregnancy (106). Conversely, NK cells from PTEN-deficient mice have hyperresponsive signaling in response to sphingosine 1-phosphate, leading to an increase in NK cells in peripheral blood as a result of aberrant trafficking to the tissue (79).

Taken together, these studies underscore the importance of PI3K signaling in NK cell development, function, and homeostasis. This importance can be further tested by studying patients with rare mutations in this pathway, particularly when these patients are considered in the context of informative mouse models.

### The Requirement for PI3K in NK Cell Lytic Function

The role of PI3K signaling in immunological synapse formation and function was first tested broadly by using relatively promiscuous inhibitors such as Ly294002 and wortmannin. These studies showed that the broad inhibition of PI3K signaling prevented NK cell cytotoxicity in NK cell lines killing *via* natural cytotoxicity and in primary cells mediating ADCC (86, 89,108, 109). While initial studies suggested that PI3K signaling wasn’t required for primary NK cell-mediated lysis of K562 targets (109), the pretreatment of IL-2-activated primary NK cells with wortmannin significantly decreases cytotoxicity against 721.221 targets, at least in part by modulating LFA-1 function (110, 111). Further studies identified PI3K–Rac1–PAK1–MEK–ERK signaling that is required for polarization and cytotoxicity of human NK cell lines and freshly isolated peripheral blood NK cells (91). Knockdown (KD) of p85α or AKT prevents lytic granule polarization to the immune synapse and inhibits the activity of Cdc42 (92). In addition, p110δ interacts directly with the SH3 domain of CrkL during NKG2D-mediated NK cell cytotoxicity and controls LFA-1-mediated conjugate formation downstream of NKG2D ligation on human NK cells (86).

Mice deficient for PI3Kδ have impaired NK cell function against tumor targets, including defects in exocytosis (75, 76). Similarly, p85α-deficient mice have impaired NK cell cytotoxicity and cytokine production (78). Specific inhibitors of PI3K class I isoforms show that while pan-PI3K inhibition impairs mature human NK cell function, the selective inhibition of PI3Kα, -β, -γ, or -δ does not have a significant effect (112). These results suggest that the genetic loss of PI3Kδ may lead to NK cell developmental defects that are not present when PI3Kδ function is inhibited in mature cells. This is supported further by studies of a mouse expressing catalytically inactive PI3Kδ, in which signaling is impaired downstream of activating receptors and NK cells fail to mature and are unable to mediate lytic function (77). Overall, however, this is a field that has been complicated by differential findings regarding the requirement for PI3K isoforms in NK cell development, in part due to differences between mouse strains (113).

While human loss-of-function mutations in PI3K110δ (68) and p85α (70) have been reported, they are overwhelmingly rare. Both defects lead to primary immunodeficiency, and NK cell number in the patient reported with p85α deficiency was significantly decreased when compared to healthy ranges (70).What have emerged as a much more common variation are GOF muta-tions in *PIK3CD* or *PIK3R1*. *PIK3CD* GOF mutations were independently reported in 2014 by Lucas et al. (65) and Angulo et al. (114) and lead to hyperactivation of PI3K110δ signaling by interrupting the interaction between PI3K110δ and the p85α-regulatory subunit, or by constitutive membrane association and activation (65, 114, 115). This hyperactivation can be detected on a cellular basis as hyperphosphorylation of S6, mTOR, and AKT. Clinically, these mutations can lead to varied phenotypes, and original *PIK3CD* cohorts were identified by screening patients with recurrent chest infections (114) and herpesviral infections (65), respectively. Patients with activating mutations in *PIK3R1* leading to an increased PI3Kδ activity also have primary immunodeficiency (67, 69, 116–123), and many studies, including large cohort studies, have defined new mutations in *PIK3CD* leading to activated PI3K delta syndrome (APDS) and expanded the phenotype of disease (64, 66, 67, 124–131). While the clinical features of patients with mutations in *PIK3CD* and *PIK3R1* can be similar, there is evidence that *PIK3R1* mutations can also have extra-immune phenotypes (118). To delineate the two where necessary, *PIK3CD* mutations are referred to as leading to APDS1, whereas mutations in *PIK3R1* cause APDS2. Finally, patients have also been identified with PTEN loss-of-function (LOF) mutations that can also lead to features of APDS (71–73).

Immunologically, APDS patients have combined immune deficiency due to impairment in multiple compartments, including B cells, T cells, and more recently described NK cells (63, 65, 114, 132). B cell function is generally impaired, and an increase in transitional B cell number is a highly conserved phenotype of APDS patients. There are also significant T cell defects in APDS patients, including increased T cell senescence likely driven by hyperproliferation, at least in some part as a result of an increased mTOR metabolism (65).

A decreased NK cell frequency has been reported in these APDS cohorts (64–67, 69); however, a detailed analysis was recently performed that identified multiple facets of deregulated NK cell phenotype and function in patients with PI3K GOF mutations (63). This includes a decreased expression of CD16 and an increased expression of CD62L in peripheral blood NK cells, suggesting incomplete terminal maturation. NK cell function is impaired, and the source of this impairment is due to multiple defects in immune synapse formation and function. These include a decreased conjugation with target cells and a decreased phosphorylation of ERK in response to activating signaling. When forming conjugates with susceptible target cells, NK cells from APDS patients have impaired MTOC polarization and actin accumulation at the immunological synapse (63). Collectively, these effects lead to impaired NK cell lytic function mediated against susceptible class I negative, as well as antibody-coated targets. These effects were demonstrated in patients with previously described E525K, E1021K, and N334K mutations, with patients with E525K mutations interestingly having a more severe impairment of NK cell function.

The treatment of patients with the mTOR inhibitor rapamycin led to improved NK cell function and partial restoration of immune synapse formation, suggesting that tonic hyperactivation of the mTOR–AKT pathway is contributing to the functional impairment of NK cells (63). The mechanism of this is currently unclear, however, and the similarity between the effect of PI3K GOF mutations and the previously reported loss-of-function models, namely impaired NK cell cytotoxicity, can be confusing. However, of note, ERK1/2 phosphorylation in response to activation was decreased in NK cells from APDS patients, counter to the expected hyperphosphorylation predicted by an increased AKT phosphorylation (and its responsiveness to rapamycin). The decreased effector function of APDS patient NK cells, combined with a decreased ERK phosphorylation, suggests that long-term hyperactivation of these pathways leads to NK cellular hyporesponsiveness. The partial reversibility of the patients’ NK cell function after the initiation of rapamycin treatment suggests that there is tunable signaling in these patient cells that can be responsive to modulation; however, this is also in addition to seemingly hardwired NK cell-developmental defects that were not affected by rapamycin treatment. There also may be differential effects of hyperactive PI3K signaling on the MAPK and AKT pathways. It would be of interest to probe downstream signalosomes with greater detail in APDS patients to determine the localization and activity of key activating and inhibitory mediators such as Vav1, SAP, and SHIP-1. In addition, while not tested in the current study, it would be of interest to test the short-term incubation of patient cells with rapamycin to determine whether NK cell function can similarly be restored by the temporary reversal of mTOR and AKT hyperphosphorylation.

Enhanced signaling through mTOR also leads to enhanced cellular metabolism, and a better understanding of how glycolysis and oxidative phosphorylation shape NK cell function is emerging (133). Consistent with the responsiveness of CD56^bright^ NK cells in response to priming, CD56^bright^ NK cells become highly metabolically active following cytokine stimulation, which enables their robust production of IFNγ. The treatment of healthy donor NK cells with rapamycin leads to a decreased production of IFNγ and a reduced expression of nutrient receptors such as the transferrin receptor (103, 134). mTor-deficient mice have impaired development due to impaired IL-15 responses, and it is likely that metabolic regulation is an important component of PI3K–mTOR-mediated IL-15 signaling and an unexplored component of the NK cell phenotype in APDS patients.

While the standard of care for many APDS patients has been rapamycin, the recent development of selective PI3Kδ inhibitors for the purpose of treating APDS patients has led to the availability of these and preliminary data about their efficacy and modes of action. The treatment of six patients with E525K or E1021K mutations with the selective PI3Kδ inhibitor leniolisib led to reduced phospho-AKT and -S6 in T cells (135, 136). While NK cells were not explicitly examined in this study, it will be of interest to determine whether, like rapamycin, specific PI3Kδ inhibition leads to restored function and whether, unlike rapamycin, this treatment also has an effect on NK cell phenotype and maturation.

### PTEN, SHIP-1, and SAP Mutations and NK Cell Function

As APDS occurs as a result of hyperactivation of the PI3K-signaling pathway, it can be informative to also consider the consequence of LOF mutations in negative regulators of PI3K signaling. Autosomal-dominant mutations in *PTEN* are a previously described cause of hamartoma tumor syndromes, with a range of clinical effects that include susceptibility to malignancy, mucocutaneous lesions, and macrocephaly (137). Whole-exome sequencing identification of patients with LOF heterozygous mutations in *PTEN* has identified patients with APDS-like characteristics (71–73). These may include many of the clinical and immunological hallmarks of APDS, including recurrent infections, lymphadenopathy, hepatosplenomegaly, and cytopenias. In addition, APDS PTEN patients had previously described features of PTEN hamartoma tumor syndrome, including macrocephaly and mental retardation. A decreased PTEN protein expression in these patients was accompanied by an increased phospho-S6 and phospho-AKT as predicted (71, 72); however, basal PtdIns(3,4,5)P3 levels were surprisingly unaffected in one patient tested (72). NK cell function in these patients has not been specifically interrogated; however, decreased NK cell numbers were reported in some PTEN patients with immune deficiency (71–73). The variable penetrance of PTEN mutations is not completely understood; however, the capacity for these mutations to phenocopy activating mutations in *PIK3CD* or *PIK3R1* speaks of the importance of modulation of this signaling pathway.

In addition, the expression and functional role of PTEN in healthy donor human NK cells has been specifically interrogated. Overexpression (OE) of PTEN in an NK cell line or primary human or transgenic mouse NK cells leads to loss of NK cell function (74). Mechanistically, this is accompanied by a decreased accumulation of F-actin at the immunological synapse and impaired granule convergence and polarization. PTEN KD in primary mouse or human cells leads to a modest increase in NK cell lytic function, underscoring the role of PTEN as a negative regulator of NK cell cytotoxicity and contrasting the effect of PI3K GOF mutations. Interestingly, unlike mouse models of SHIP-1 and human patients with APDS, in which deregulation of this pathway impairs NK cell maturation, there was no reported effect of PTEN OE on NK cell development in the transgenic super-PTEN mouse model. By contrast, SHIP-1-deficient mice have reduced NK cell numbers in the periphery, specifically due to impaired terminal maturation from immature precursors (80). In human NK cells, both SHIP-1 and PTEN have differential expression in mature subsets, with PTEN being highly expressed in CD56^bright^ NK cells (74) and SHIP-1 more highly expressed in the CD56^dim^ subset (138). SHIP-1 additionally plays a role in modulating signaling downstream of CD16 as it is recruited to the TCRζ chain during ADCC and can negatively regulate cytotoxicity (139).

Finally, the activating receptor 2B4 associates with SAP/SH2D1A, an SH2-domain-containing adaptor molecule. In addition to other immune defects, patients with X-linked lymphoproliferative disease (XLP) as a result of mutations in SAP have impaired NK cell function (140). The inhibition of PI3K function disrupts the 2B4–SAP interaction, and conversely PI3K function is impaired in patients with SAP mutations (82). As a result, the treatment of NK cells from patients with XLP with PI3K inhibitors does not further affect NK cell-cytotoxic function, whereas in healthy donors, PI3K inhibition impairs NK cell lytic function.

### NK Cell Maturation and Homing

The role of PI3K in cytokine signaling, as well as its role in Rac signaling and actin remodeling, points strongly to a critical role in governing NK cell migration and maturation. In addition, PI3Kγ mediates migration through its association with G-protein-coupled receptors, and p110α and p110δ play a role in lymphocyte chemotaxis and migration. Pan-PI3K inhibition reduces NK cell migration in response to chemokines, and the selective inhibition of p110γ or p110δ shows that both play a role in CXCL12-mediated NK cell migration (106). The deletion of the p110δ isoform in mice leads to a significant decrease in NK cell number in peripheral organs, with distinct phenotypic abnormalities including a decreased expression of Ly49G2, Ly49C/I, and CD11b/CD43 (75). In remaining cells, cytokine secretion, but not production, is impaired, demonstrating a role for p110δ in both maturation and function. Transgenic mice expressing catalytically inactive p110δ also have reduced numbers of mature NK cells in the periphery, although NK cell progenitors in bone marrow are present at normal frequency (77). As with p110δ-deficient mice, catalytically inactive p110δ mice have a decreased frequency of inhibitory Ly49C/I-positive NK cells, indicating impaired terminal maturation or receptor regulation. Discrepancies between isoform-specific knockout mice and catalytically inactive mutants may be due to altered expression of other PI3K subunits following single isoform deletion, including altered p85α, p110β, and p110γ expression in p110δ-knockout mice (141, 142). PTEN also plays a critical role in NK cell homing, and NK cell-specific PTEN deletion in mice leads to increased NK cell numbers in peripheral blood due to premature egress from the bone marrow and altered response to S1P signals (79).

Whether NK cells from patients with activating PI3K mutations have alterations in NK cell maturation, tissue distribution, homeostasis, or migration is not fully understood. A direct correlation to the effect seen with PTEN deletion in mice is not seen, as abnormal NK cell numbers in peripheral blood of APDS patients have been reported as decreased, not increased (63–67, 69). Despite a decreased NK cell number, the ratio of CD56^bright^ to CD56^dim^ NK cells is not significantly affected in these patients, suggesting that terminal maturation occurs (63). However, there are receptors that are associated with NK cell development that have significantly altered expression in APDS patient NK cells. These include a decreased expression of CD16 on CD56^dim^ NK cells and a decreased expression of CD62L on CD56^bright^ NK cells. Altered expression of CD62L could lead to impaired localization in secondary lymphoid tissue, and PTEN knockout mice also have a significantly decreased CD62L expression (79). Notably, NK cells from APDS patients had a significantly decreased expression of CD122, the common gamma chain, and CD127, the IL-7 receptor. Finally, an increased expression of NKG2A on CD56^dim^ NK cells from APDS patients is also suggestive of dysregulated maturation. Taken together, however, it is difficult to interpret these selective receptor anomalies with a cohesive defect in a specific aspect of NK cell maturation. Additional studies, including gene expression and, if possible, the study of tissues beyond peripheral blood, are required to definitively isolate defects in NK cell maturation and homing in patients with activating *PIK3CD* mutations.

### What Can Patients with PI3K Mutations Teach Us about NK Cell Function?

**Figure 1:**
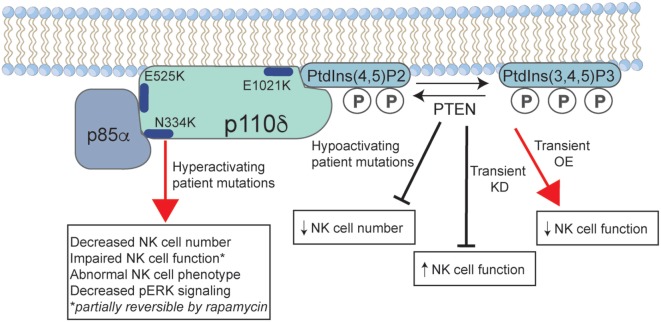
The effect of activated PI3K delta syndrome (APDS)-causing mutations on human natural killer (NK) cell function. Common mutations in p110δ that lead to disease are shown, including those that lead to loss of negative regulation by the p85α-regulatory subunit (E525K, N334K), and the E1021K mutation that leads to constitutive membrane association. Phosphoinositide-3-kinase (PI3K) p110δ catalyzes the conversion of PtdIns(3,4)P2 to PtdIns(3,4,5)P3 at the cell membrane, a reaction that is negatively regulated by phosphatase and tensin homolog (PTEN). Gain-of-function mutations in PI3Kδ lead to a decreased NK cell number and aberrant phenotype. While less well described, patients with loss-of-function mutations in PTEN may have an APDS phenotype that is accompanied by a decreased NK cell number. Studies of short-term KD or OE of PTEN in human NK cells lead to increased and decreased NK cell functions, respectively. P, phosphate; KD, knockdown; OE, overexpression.

What can we learn about the requirement for, and role of, PI3K signaling in human NK cells from patients with primary immunodeficiency? The discovery of APDS provides us with a robust number of patients to study and underscore the biological complexity of this signaling pathway. While these mutations are termed GOF, and rightly so based upon the hyperphosphorylation of key signaling molecules, they are not represented by an increased NK cell function. This can be partially explained by signs of impaired activation of signaling intermediates such as phospho-ERK and phospho-JNK (63), suggesting that NK cell hyporesponsiveness may result from constitutive overactivation of the pathway. This apparent hyporesponsiveness is also in contrast to studies of human cells in which PTEN levels have been transiently manipulated, with KD of PTEN leading to an increased NK cell function in this system, as would be predicted by the loss of inhibition of the PI3K-signaling pathway (Figure 1) (74). The mechanism of hyporesponsiveness in APDS patient NK cells is unclear, but the restoration of function following rapamycin treatment suggests that this may be a reversible condition. Currently, however, we do not know whether the effect of rapamycin is on long-term NK cell development or on survival, or whether short-term rapamycin treatment would lead to similar regained function. Treatment of patients with rapamycin may lead to the replenishment over time of NK cell subsets that have developed with modulated IL-15–mTOR signaling, leading to the restoration of functional capacity. Given the particular susceptibility of these patients to herpesviral infection (including Epstein–Barr virus, cytomegalovirus, and varicella zoster), it is likely that their NK cell dysfunction contributes to this clinical phenotype.

It is also important to consider the overall immune environment in these patients. Deregulation of the B and T cell subsets may additionally affect the generation or homeostasis of NK cell subsets, through direct or indirect mechanisms. It will be of value to study the NK cells of these patients more closely to better determine the molecular basis of dysfunction. In addition, a mouse model of APDS would enable the further dissection of the effect of activated PI3K on NK cell development, migration, and cytotoxicity. Better understanding of the effect of these mutations specifically on NK cells will be important for better understanding and implementing the next generation of therapies, including targeted small molecule inhibitors. As always in the case of primary immunodeficiency, these patients also provide us the rare opportunity to better understand the requirements for human immunity through the study of a uniquely human model.

## Author Contributions

All the work was performed by EM.

## Conflict of Interest Statement

The author declares that the research was conducted in the absence of any commercial or financial relationships that could be construed as a potential conflict of interest. The reviewer TP and the handling editor declared their shared affiliation.
